# Myocardial work efficiency in children with Wolff-Parkinson-White preexcitation before and after accessory pathway ablation

**DOI:** 10.3389/fcvm.2026.1812719

**Published:** 2026-05-29

**Authors:** Tereza Hadžić, Olena Iurchenko, Jan Kovanda, Viktor Tomek, Terézia Tavačová, Peter Kubuš, Jan Janoušek

**Affiliations:** Children's Heart Centre, 2nd Faculty of Medicine, Charles University and Motol and Homolka University Hospital, Prague, Czechia

**Keywords:** ablation, myocardial work, pediatric cardiology, speckle-tracking echocardiography, Wolff-Parkinson-White syndrome

## Abstract

**Background and objective:**

Ventricular preexcitation alters the electromechanical activation sequence and may induce left ventricular (LV) contractile discoordination and pathologic remodeling. We aimed to evaluate LV myocardial work (MW) before and after accessory pathway (AP) ablation and to compare it to controls in a prospective observational study.

**Methods:**

MW calculation was performed in 37 pediatric patients with Wolff-Parkinson-White preexcitation before and within 24 h after catheter ablation using a commercial software. MW was calculated as the area under pressure-strain loop derived from speckle tracking echocardiography and blood pressure. Thirty healthy age- and weight-matched individuals were used as controls.

**Results:**

Before ablation LV ejection fraction (mean 55.3 in patients vs. 59.3% in controls, *P* = 0.005) and global LV MW efficiency (median 93.0 vs. 95.0%, *P* < 0.001) were lower in patients vs. controls. Global LV MW efficiency correlated negatively with QRS duration (R = 0.519, *P* < 0.001) and the interval between earliest delta wave onset and R wave peak in lead V6 (R = 0.705, *P* < 0.001), respectively. Segmental LV MW efficiency increased from median 93.0% at AP location to 97.0% in distant segments (*P* < 0.001). After successful ablation (*N* = 31/37 patients) global LV MW efficiency increased from mean 91.8 to 93.6% (*P* = 0.013) and segmental efficiency was more equally distributed (*P* < 0.001) with increase in segments adjacent to ventricular AP insertion (*P* = 0.006).

**Conclusion:**

Ventricular preexcitation is associated with decreased LV MW efficiency which correlates negatively with the degree of preexcitation and improves early after ablation. Wasted work in asymptomatic preexcitation may be considered when assessing the potential for pathologic LV remodeling or discussing the indication for prophylactic ablation.

## Graphical Abstract

**Figure:**
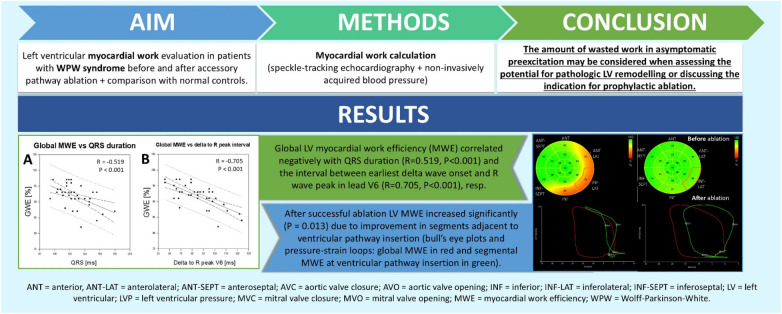

## 1 Introduction

Ventricular preexcitation, as observed in patients with Wolff-Parkinson-White (WPW) syndrome or pattern, alters the ventricular electromechanical activation sequence and may induce left ventricular (LV) contractile discoordination and in some cases pathologic LV remodeling (1, 2). The typical picture which can be non-invasively assessed by speckle-tracking echocardiography includes early contraction of segments adjacent to the ventricular accessory pathway (AP) insertion associated with a pre-stretch of segments located remotely from the AP (1). Disparities in segmental myocardial preload as well as myocardial work (MW) are a consequence.

In 2012, Russel et al. suggested a new method of non-invasive calculation of MW, which combines speckle-tracking echocardiography and blood pressure (3). This method follows the basic principles of MW calculation described in earlier studies (4), includes the afterload changes in MW calculation and allows to assess MW both globally and regionally, which can be useful in patients with local contractile abnormality (5, 6).

The aim of our study was to evaluate acute changes in LV MW before and after AP ablation in young patients with WPW preexcitation, to compare them to normal controls and to quantify the negative impact of ventricular preexcitation on myocardial work efficiency (MWE). The main hypothesis of this study was that both global and segmental MWE is decreased in ventricular preexcitation and improves already early after AP ablation.

## 2 Methods

### 2.1 Study population

**Figure 1:**
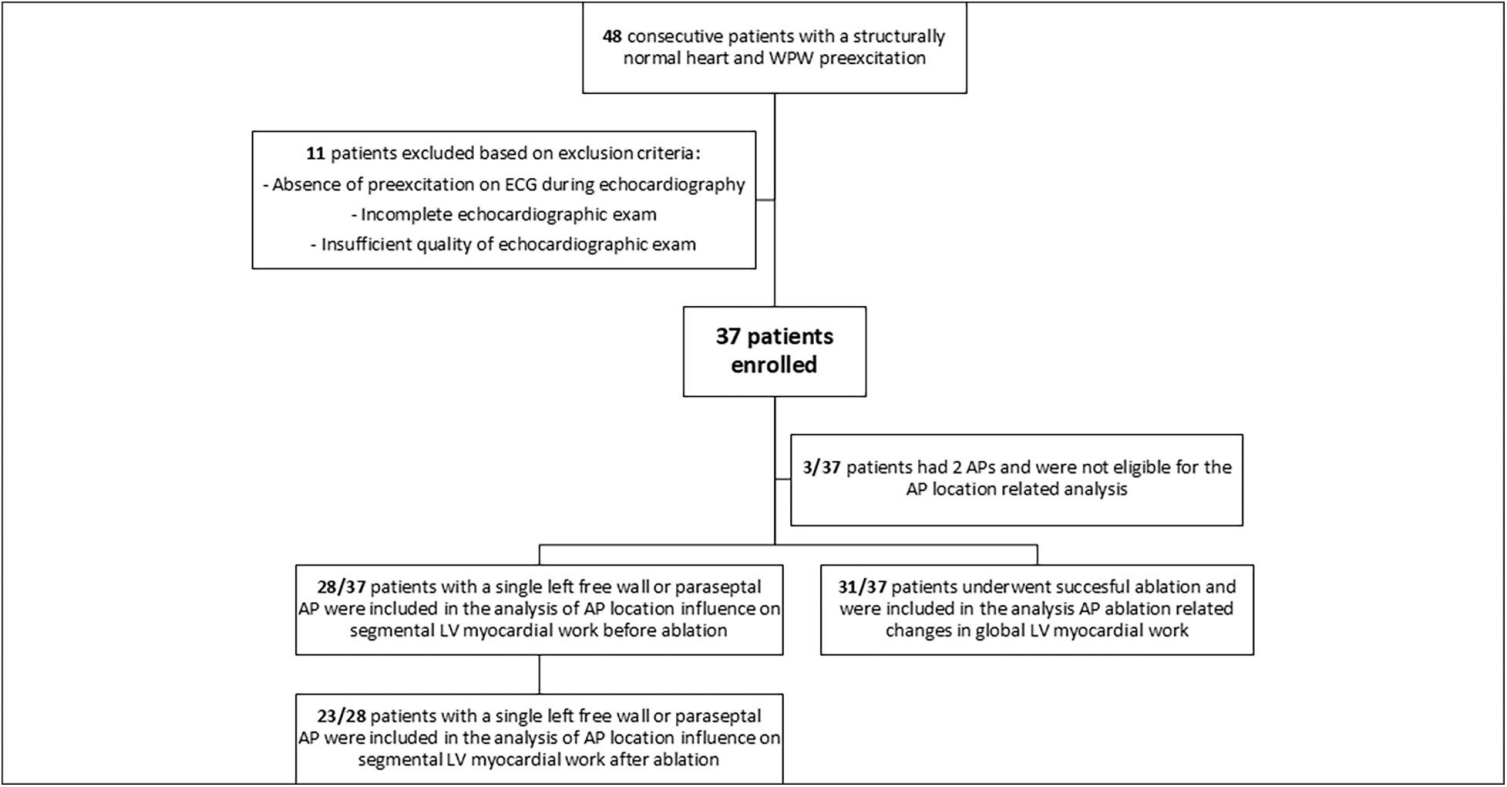
A flowchart of the enrollment process. AP,  accessory pathway; LV,  left ventricular; WPW, Wolff-Parkinson-White.

Over an interval of one year forty-eight patients with a structurally normal heart and WPW preexcitation scheduled for electrophysiological study and catheter ablation were prospectively enrolled (see Figure 1) and underwent MW evaluation. During the process, 11 patients were excluded for either absence of preexcitation on ECG during echocardiographic exam or incompleteness or insufficiently low image quality (to perform MW analysis) of echocardiographic exam. Thirty-seven patients were therefore ultimately enrolled in the presented observational prospective study. Three of the 37 patients had two accessory pathways and were not eligible for the analysis of the influence of AP location on echocardiographic parameters. Thirty-one of the 37 patients underwent successful AP ablation and were included in the analysis of AP ablation effect. Finally, 28 patients with a single left free wall or paraseptal AP before ablation and 23 corresponding patients after successful ablation could be included in the analysis of the relationship between LV segmental proximity to ventricular AP insertion and segmental echocardiographic data, see further. Post-procedural echocardiography was performed 16–24 h after ablation before discharge as part of the routine clinical management. Sex of the participating patients was not taken into account, hence the differences in results between sexes were not evaluated. During the same interval, thirty age-, weight- and sex-matched individuals with a normal electrocardiogram (ECG) without preexcitation or a history of recent tachycardia and with a structurally normal heart and function (see Tables 1 and 2) were evaluated as controls before scheduled ablation of a paroxysmal supraventricular tachycardia (atrioventricular nodal reentrant tachycardia in 18, atrioventricular tachycardia due to a concealed AP in 9, Mahaim fascicle related atrioventricular tachycardia in 1 and structurally and functionally normal heart in 2). None of the patients and 4/30 control group subjects were on antiarrhythmic medication (Sotalol in all) before electrophysiological study. The medication was discontinued for 5 half-lives before admission to minimize the effect of drug treatment on pre-ablation evaluation.

**Table 1:** Patient and controls characteristics.

Parameter	WPW patients	Normal controls	*p*-value	Power of the test
Number	37	30	-	-
Sex [N] *(%)*^a^				
- Female - Male	13 (35) 24 (65)	9 (30) 21 (70)	0.795	N/A
Age [years] *median (IQR)*	13.2 (11.5–16.2)	15.2 (10.5–16.9)	0.627	N/A
Height [cm] *mean (SD)*	163.0 (15.7)	160,0 (21.4)	0.505	0.101
Weight [kg] *median (IQR)*	56.9 (43.3–66.0)	57.5 (31.8–66.9)	0.442	N/A
Body surface area [m2] *median (IQR)*	1.57 (1.37–1.78)	1.61 (1.11–1.83)	0.719	N/A
QRS duration [ms] *median (IQR)*	122 (115–138)	87 (81–93)	<0.001	N/A
Accessory pathways *Paraseptal* *Left free wall* *Right free wall*	[N] = 40^b^ 20 14 6	-	-	-

a The data about sex were acquired from existing medical documentation.

b 3/37 patients had 2 accessory pathways.

IQR, inter-quartile range; N, number; SD, standard deviation; WPW, Wolff-Parkinson-White.

**Table 2:** Baseline values in WPW patients vs. controls.

Parameter	WPW patients	Normal controls	*p*-value	Power of the test
Number	37	30	-	-
LV EF [%] *mean (SD)*	55.3 (6.1)	59.3 (4.8)	0.005	0.824
Global longitudinal LV strain [%] *median (IQR)*	−19.0 (−21.2 – −17.1)	−20.0 (−21.0 – −18.0)	0.590	N/A
Peak longitudinal LV strain dispersion [ms] *median (IQR)*	39.3 (33.6–45.1)	33.1 (29.2–35.5)	<0.001	N/A
Global LV MWI [%mmHg] *mean (SD)*	1,801.6 (367.3)	1,771.9 (306.7)	0.725	0.050
Global LV MCW [%mmHg] *mean (SD)*	2,190.2 (400.5)	2,171.9 (321.7)	0.840	0.055
Global LV MWW [%mmHg] *median (IQR)*	164.0 (108.0–219.5)	97.0 (81.5–115.5)	<0.001	N/A
Global LV MWE [%] *median (IQR)*	93.0 (90.5–94.0)	95.0 (94.0–96.0)	<0.001	N/A

EF, ejection fraction; IQR, inter-quartile range; LV, left ventricle/ventricular; MCW, myocardial constructive work; MWI, myocardial work index; MWW, myocardial wasted work; SD, standard deviation; WPW, Wolff-Parkinson-White.

### 2.2 12-lead electrocardiogram

Standard 12-lead ECG was recorded and maximum QRS duration in any lead as well as the interval between the earliest delta wave onset and R wave peak in lead V6 were measured using a digital ECG analysis platform (CardioPoint®, BTL Industries, Prague, Czech Republic) as a surrogate for the magnitude of preexcitation.

### 2.3 Echocardiographic examination

**Figure 2:**
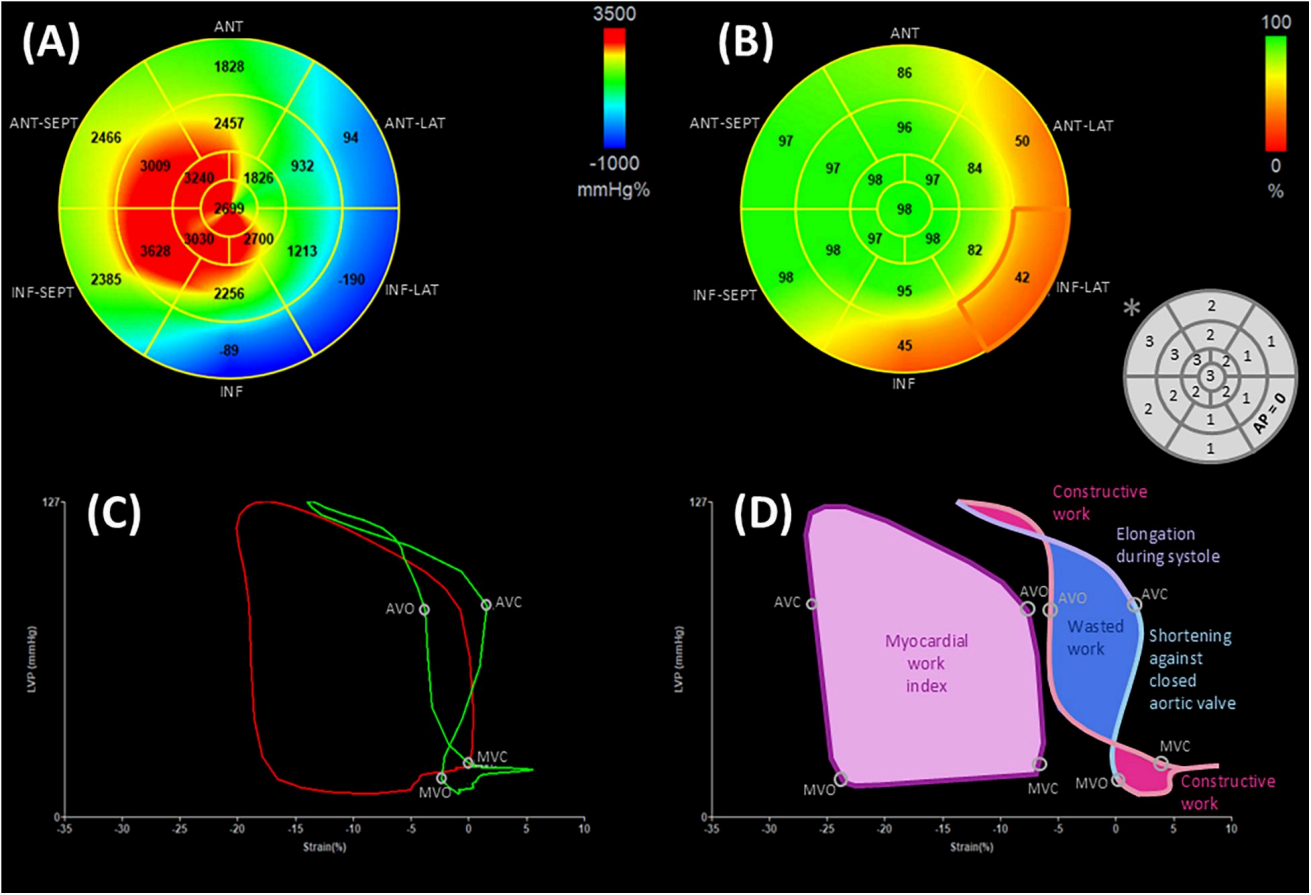
Myocardial work analysis in a patient with WPW syndrome and left inferolateral AP. The bull's eye plot represents LV myocardium divided into individual segments and shows: **(A)** Segmental values of MWI in mmHg%; **(B)** Values of MWE in %; **(C)** Global LV pressure-strain loop (red) and pressure-strain loop representing the segment at ventricular AP insertion (green), where pressure-strain loop area from mitral valve closure to mitral valve opening stands for performed myocardial work, as explained further in the text (see Methods). Major decrease of both MWI and MWE along with a distorted pressure-strain loop is evident in segments adjacent to AP. **(D)** Graphical presentation of the methodology of MWI, MCW and MWW calculations (30). *Annotation of segmental distance from ventricular accessory pathway AP insertion. ANT, anterior; ANT-LAT, anterolateral; ANT-SEPT, anteroseptal; AP, accessory pathway; AVC, aortic valve closure; AVO, aortic valve opening; INF, inferior; INF-LAT, inferolateral; INF-SEPT, inferoseptal; LV, left ventricular; LVP, left ventricular pressure; MVC, mitral valve closure; MVO, mitral valve opening; MWE, myocardial work efficiency; MWI, myocardial work index; WPW, Wolff-Parkinson-White. Figure 2 consists of screenshots from the EchoPAC platform (see the Echocardiographic examination, Methods section) and graphics created by the first author.

Echocardiographic evaluation was performed before and 16–24 h after catheter ablation according to standard clinical care procedure using the Vivid E95 equipment (GE-Vingmed, Horten, Norway). 2D loops of 3 cardiac cycles were obtained along with ECG in three apical views (4-chamber, 2-chamber and apical long axis view) to enable offline analysis of MW by the GE EchoPAC^TM^ platform with the integrated Automated Function Imaging Module (AFI, software version 204.57.0.745). The frame rate for speckle tracking data acquisition ranged from a minimum 46.1 to a maximum of 87.6 frames per second, with careful focus on image quality and ventricular wall visualization (5, 7). Median (inter-quartile range) frame rates at the pre- and post-ablation evaluation displayed changes which were regarded methodologically insignificant: apical 4-chamber view 61.5 (55.2–62.2) vs. 61.8 (57.2–62.2) frames per second, P NS; apical 2-chamber view 62.3 (57.7–62.7) vs. 61.8 (57.1–62.2) frames per second, *P* = 0.012; apical long axis view 62.2 (57.7–62.7) vs. 61.7 (57.1–62.3) frames per second, *P* = 0.034. LV ejection fraction (EF) was measured using the biplane Simpson's method. Images were saved for later offline analysis using the EchoPAC workstation according to described methodology (3). In brief, longitudinal strain speckle-tracking LV images obtained in three apical views were analyzed by the EchoPAC AFI module with automatic wall detection and manual adjustment if necessary (5–7) and segmental strain curves were constructed by the software. Time zero of the strain curves is automatically set on the R wave of QRS complex by the software. Subsequently, timing of mitral and aortic valve opening and closure was manually adjusted. Patient's blood pressure was acquired in a standard manner using a brachial cuff immediately before echocardiography (5, 6). Both global and segmental MW indices were calculated and saved for further analysis. These included global and segmental LV myocardial work index (MWI), constructive work (MCW), wasted work (MWW) and myocardial work efficiency (MWE) (5, 6). Corresponding bull's eye plots showed values for 18 individual segments of the LV myocardium (6 basal, mid-ventricular and apical segments, respectively) using a variant of standardized LV standardized LV segmentation (8) adopted by the AFI software. Respective global and segmental pressure-strain loops were also constructed (Figure 2). Myocardial work index was calculated as the area of the pressure-strain loop from mitral valve closure to mitral valve opening. MCW was defined as total work performed by myocytes that shorten during systole and elongate during isovolumic relaxation and contribute to LV ejection. MWW was defined as work performed by myocytes not contributing to pump function with lengthening during systole and shortening against closed aortic valve (Figure 2D). MWE was calculated as the ratio of MCW to the sum of MCW and MWW (5, 6). Additionally, global longitudinal LV strain as well as peak longitudinal strain dispersion (= standard deviation of time to peak segmental strain) were measured and calculated from the 18 LV segments by the AFI software. Adequate quality of the speckle-tracking data in all three LV projections to allow for analysis of all LV segments was required for inclusion into the study. Manual adjustments were performed, if necessary, mainly in case of strain double peaks caused by artifacts. The echocardiographers performing the evaluation were not blinded to the ECG analysis and AP ablation results as this would be difficult to guarantee in the daily clinical routine.

### 2.4 Electro-anatomical mapping

All antiarrhythmic drugs were discontinued for 5 half-lives before admission to minimize the effect of drug treatment on pre-ablation evaluation. 3D electro-anatomical mapping using the Ensite Precision^TM^ system (Abbott Cardiovascular, Plymouth, Minnesota, USA) was used to localize the ventricular AP insertion during electrophysiological study in patients with WPW preexcitation before ablation to one of the following basal septal or LV free wall segments according to standardized echocardiographic segmentation (8): anteroseptal, inferoseptal, inferior, inferolateral, anterolateral and anterior. In case of right free wall APs the respective assigned locations were: anterior, anterolateral, lateral, inferolateral and inferior. For further analysis, segmental distance from the ventricular AP insertion to any of the 18 LV segments was calculated in case of LV free wall and paraseptal pathways applying the following rules: Basal segment corresponding to ventricular AP insertion was assigned as 0, all neighboring segments as 1, segments with one interposed segment as 2 and those with 2 interposed segments as 3 (Figure 2*).

### 2.5 Statistics

Continuous variables were summarized either as median (interquartile range, IQR) or as mean (standard deviation, SD) depending on the pattern of distribution, whereas categorical variables were summarized as absolute numbers and relative frequencies. Fisher exact test was used to determine the difference in relative frequencies between two groups. Inter-group comparisons of continuous variables were executed through the unpaired t-test or the Mann–Whitney rank sum test as appropriate. Intra-group comparisons were analyzed using the paired t-test or Wilcoxon signed rank test. Kruskal–Wallis One Way Analysis of Variance on Ranks was applied to compare three and more unrelated groups of continuous variables. Linear regression was used to analyze the relationship between two continuous variables. Multiple backward stepwise regression was applied to evaluate the influence of two and more independent variables in case of a univariate *p*-value <0.05. Inter-observer coefficient of variance and the Bland-Altman plot was used to assess the inter-observer agreement of MW parameters measurements in 10 randomly selected patients before and after AP ablation with the help of a second observer. Measurements conducted solely by Observer No 1 were used for all remaining analyses. Differences between sexes were not evaluated because of the relatively small sample size. SigmaPlot version 15.0 was used for all aforementioned statistical work-up with significance level set to *p* < 0.05. The power of the performed tests is displayed whenever available.

### 2.6 Consent

Because of the absence of any impact on clinical patient management and anonymized data presentation, a specific informed consent for the conduction of the study was not required. An informed consent was, however, required for the catheter ablation procedure and related clinical evaluations as part of the hospital standards.

## 3 Results

### 3.1 General characteristics of participants

**Figure 3:**
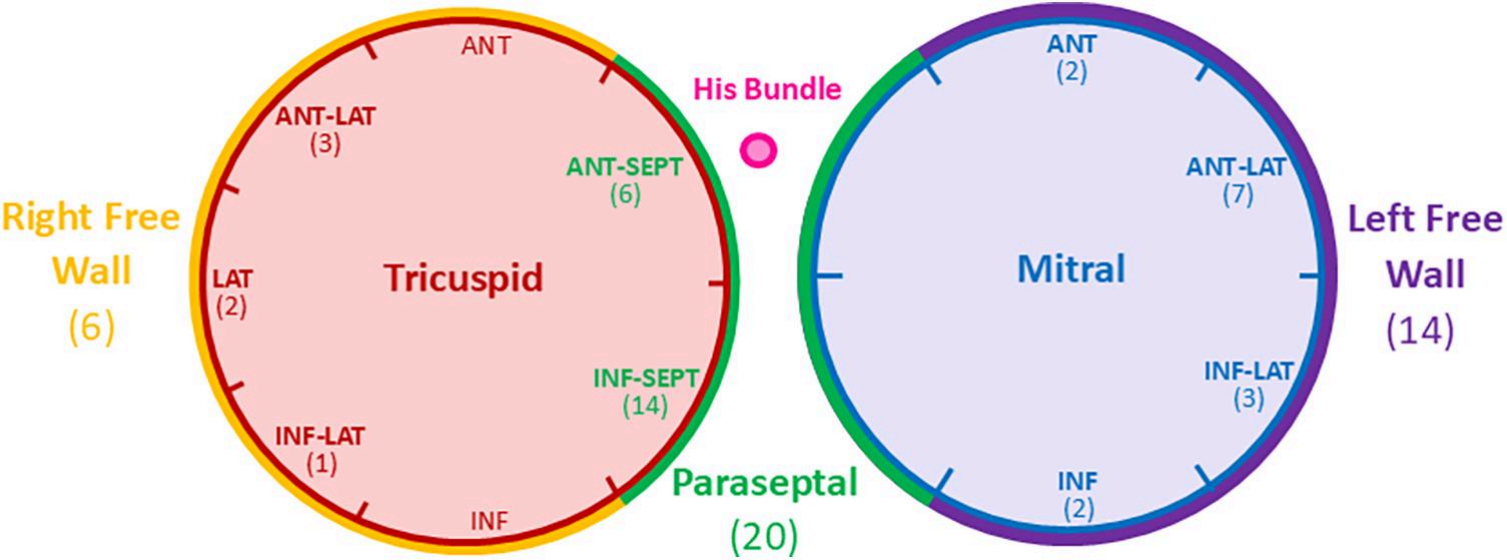
Accessory pathway location. The numbers in parentheses indicate the count of accessory pathways in each location as they occurred in the study population. ANT, anterior; ANT-LAT, anterolateral; ANT-SEPT, anteroseptal; INF, inferior; INF-LAT, inferolateral; INF-SEPT, inferoseptal; LAT, lateral. Figure 3 was created by the first author.

Baseline (before ablation) study population characteristics are given in Table 1. Demographic parameters did not differ between patients and controls. Distribution of AP locations is displayed in Figure 3.

### 3.2 WPW patients vs. controls

LV ejection fraction and global LV MWE were both lower and peak longitudinal LV strain dispersion as well as the global LV MWW were higher in WPW patients at baseline as compared to controls. Other measured parameters did not differ (Table 2).

### 3.3 Degree of preexcitation

**Figure 4:**
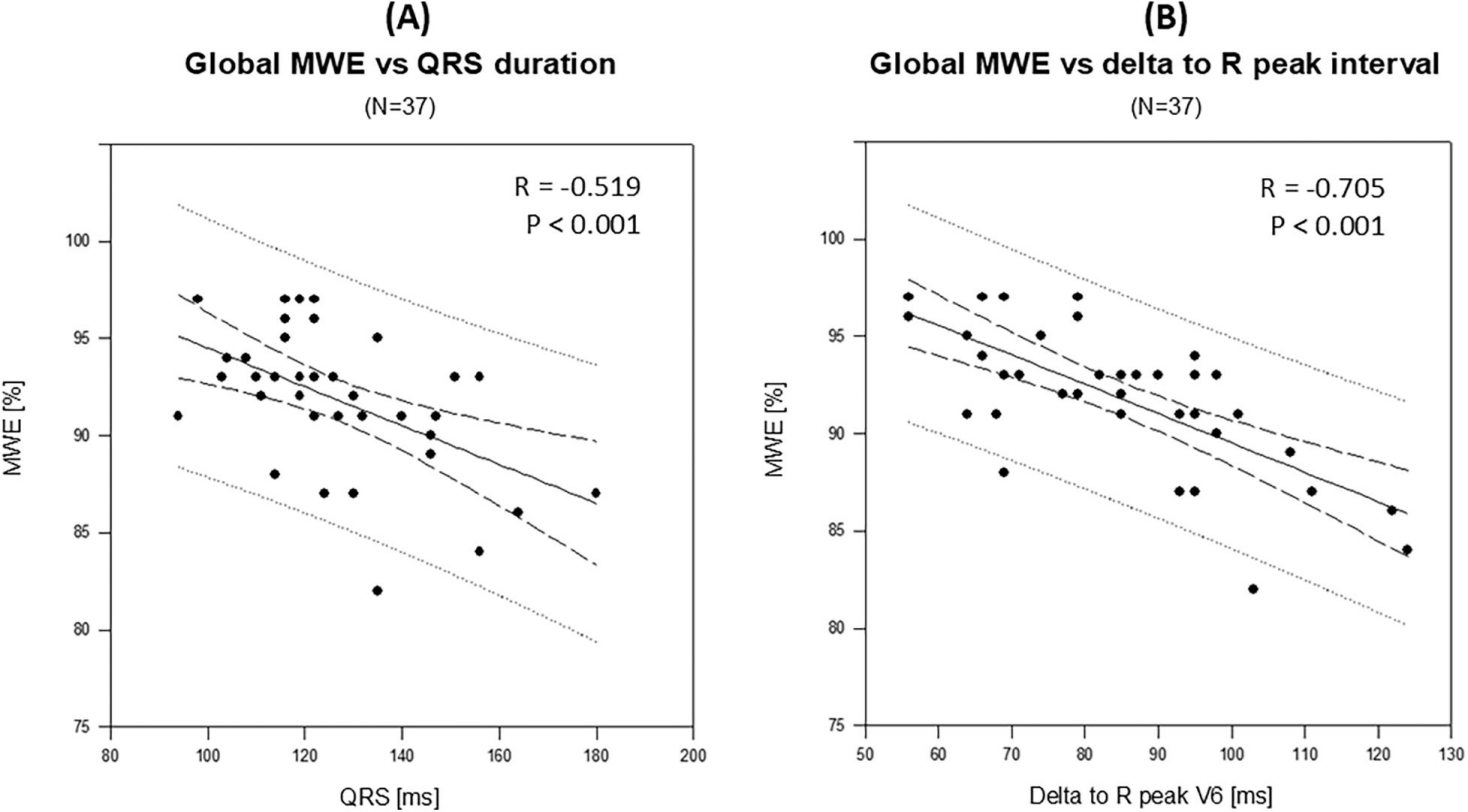
Global myocardial work efficiency and the degree of preexcitation in patients with WPW preexcitation before ablation. The degree of preexcitation was expressed as the duration of QRS complex and the interval from the earliest delta wave onset to R wave peak in V6. Relationship between the two variables was evaluated using linear regression A. MWE vs. QRS duration. B. MWE vs. delta to R peak interval in V6. LV,  left ventricular; MWE, myocardial work efficiency; WPW, Wolff-Parkinson-White. Figure 4 was created in the SigmaPlot statistical software (see the statistics section), labels were added in MS PowerPoint.

With increasing degree of preexcitation, both LV contractility (as measured by global longitudinal LV strain) and LV MWE decreased, whereas both peak longitudinal strain dispersion and global LV MWW increased - reflecting more contractile LV discoordination (Table 3, Figure 4).

**Table 3:** LV mechanics, myocardial work and the degree of preexcitation.

Parameter	QRS duration	Power of the test	Delta to peak R in V6	Power of the test
LV EF	R = −0.187, P = 0.268	0.196	R = −0.268, P = 0.109	0.360
Global longitudinal LV strain	R = + 0.313, P = 0.059	0.471	R = + 0.483, P = 0.002	0.868
Peak longitudinal LV strain dispersion	R = + 0.311, P = 0.194	0.253	R = + 0.426, P = 0.01	0.743
Global LV MWI	R = −0.200, P = 0.235	0.219	R = −0.372, P = 0.023	0.625
Global LV MCW	R = −0.064, P = 0.707	0.056	R = −0.197, P = 0.243	0.213
Global LV MWW	R = + 0.453, P = 0.005	0.814	R = + 0.632, P < 0.001	0.991
Global LV MWE	R = −0.519, *P* < 0.001	0.918	R = −0.705, *P* < 0.001	0.999

EF, ejection fraction; LV, left ventricle/ventricular; MCW, myocardial constructive work; MWE, myocardial work efficiency; MWI, myocardial work index; MWW, myocardial wasted work.

### 3.4 Accessory pathway location

In patients with a single AP (*N* = 34) peak longitudinal strain dispersion was the only parameter statistically different between patients with the left free wall, paraseptal and right free wall APs being the highest in the latter. In a stepwise regression, however, the delta to R peak interval remained the only significant predictor (*P* = 0.008) of peak longitudinal strain dispersion. Other LV mechanics and MW parameters were not significantly influenced by AP location (Table 4).

**Table 4:** LV mechanics, myocardial work and the accessory pathway location^a^.

Parameter	Accessory Pathway Location				
	Left free wall (*N* = 10)	Right free wall (*N* = 6)	Paraseptal (*N* = 18)	*p*-value	Power of the test
QRS [ms] *mean (SD)*	124.3 (28.0)	131.5 (18.5)	125.9 (13.2)	0.766	N/A
Delta to peak R in V6 [ms] *mean (SD)*	83.6 (21.1)	95.7 (18.4)	82.3 (14.4)	0.259	0.111
LV EF [%] *mean (SD)*	53.8 (6.4)	57.7 (3.6)	55.6 (6.8)	0.490	N/A
Global longitudinal LV strain [%] *mean (SD)*	−19.0 (2.9)	−17.8 (2.3)	−19.7 (2.5)	0.327	0.073
Peak longitudinal LV strain dispersion [ms] *mean (SD)*	36.8 (6.1)	49.3 (7.4)	39.3 (7.9)	0.007	0.764
Global LV MCW [%mmHg] *mean (SD)*	2,407.6 (501.9)	2,027.8 (380.9)	2,109.3 (341.9)	0.115	0.264
Global LV MWW [%mmHg] *median (IQR)*	133.5 (71.3–254.8)	183.0 (1,74.0–241.3)	141.5 (99.8–189.0)	0.247	N/A
Global LV MWE [%] *mean (SD)*	92.8 (3.9)	89.5 (3.6)	91.9 (3.7)	0.235	0.127

a in patients with a single accessory pathway (*N* = 34/37).

EF, ejection fraction; LV, left ventricular; MCW, myocardial constructive work; MWE, myocardial work efficiency; MWW, myocardial wasted work; SD, standard deviation;.

### 3.5 WPW patients before and after ablation

**Figure 5:**
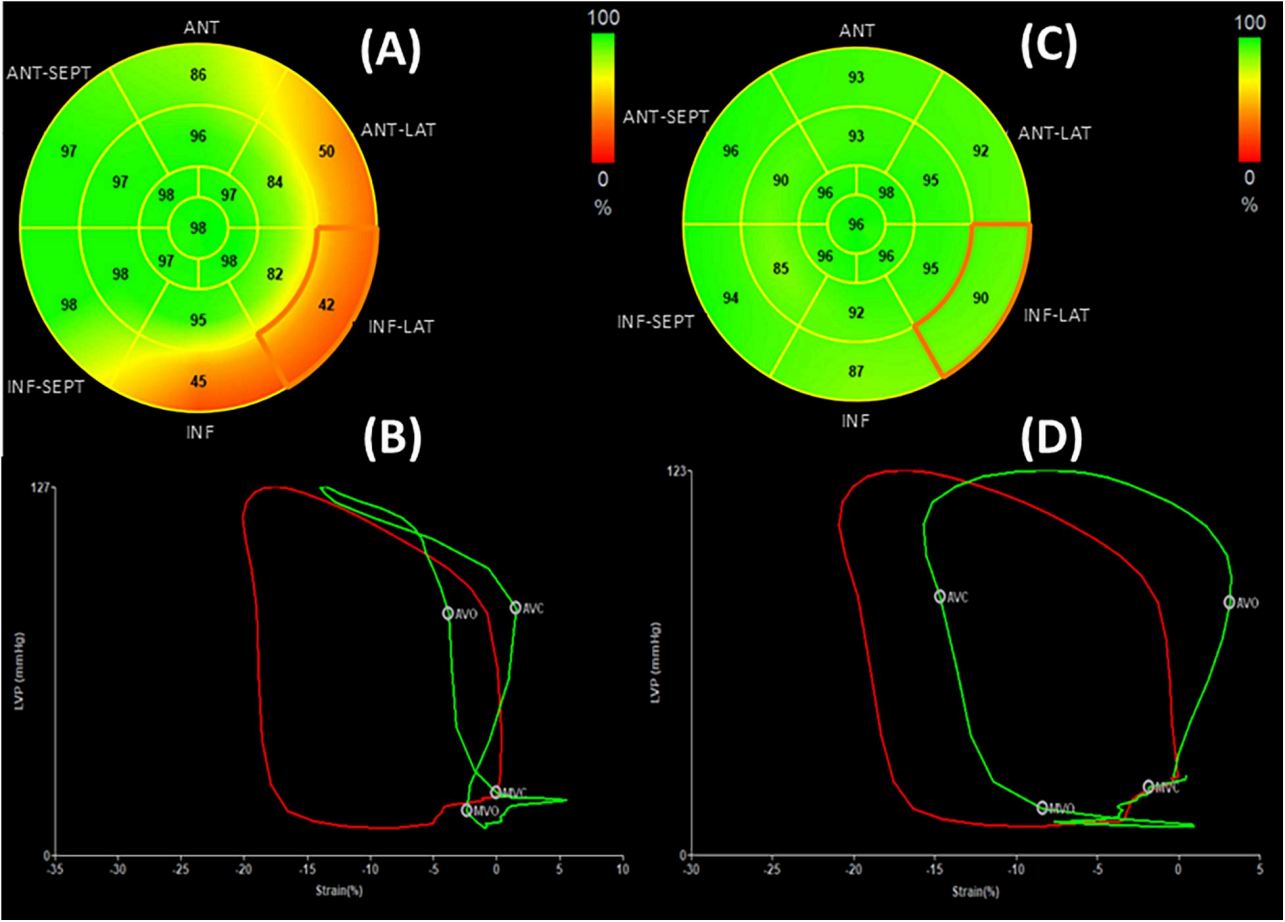
Segmental data before and after ablation of left inferolateral AP. Myocardial work efficiency, global LV pressure-strain loop (red) and segmental pressure-strain loop from the ventricular AP insertion site (green) before **(A)** and **(B)** and after **(C)** and **(D)** AP ablation. The area under the pressure-strain loop corresponds to the MWI. Normalization of both the segmental loops and segmental MWE at the AP insertion is seen after ablation. ANT, anterior; ANT-LAT, anterolateral; ANT-SEPT, anteroseptal; AP, accessory pathway; AVC, aortic valve closure; AVO, aortic valve opening; INF, inferior; INF-LAT, inferolateral; INF-SEPT, inferoseptal; LV, left ventricular; LVP, left ventricular pressure; MVC, mitral valve closure; MVO, mitral valve opening; MWE, myocardial work efficiency; MWI, myocardial work index. Figure 5 consists of screenshots from the EchoPAC platform (see the echocardiography section).

**Figure 6:**
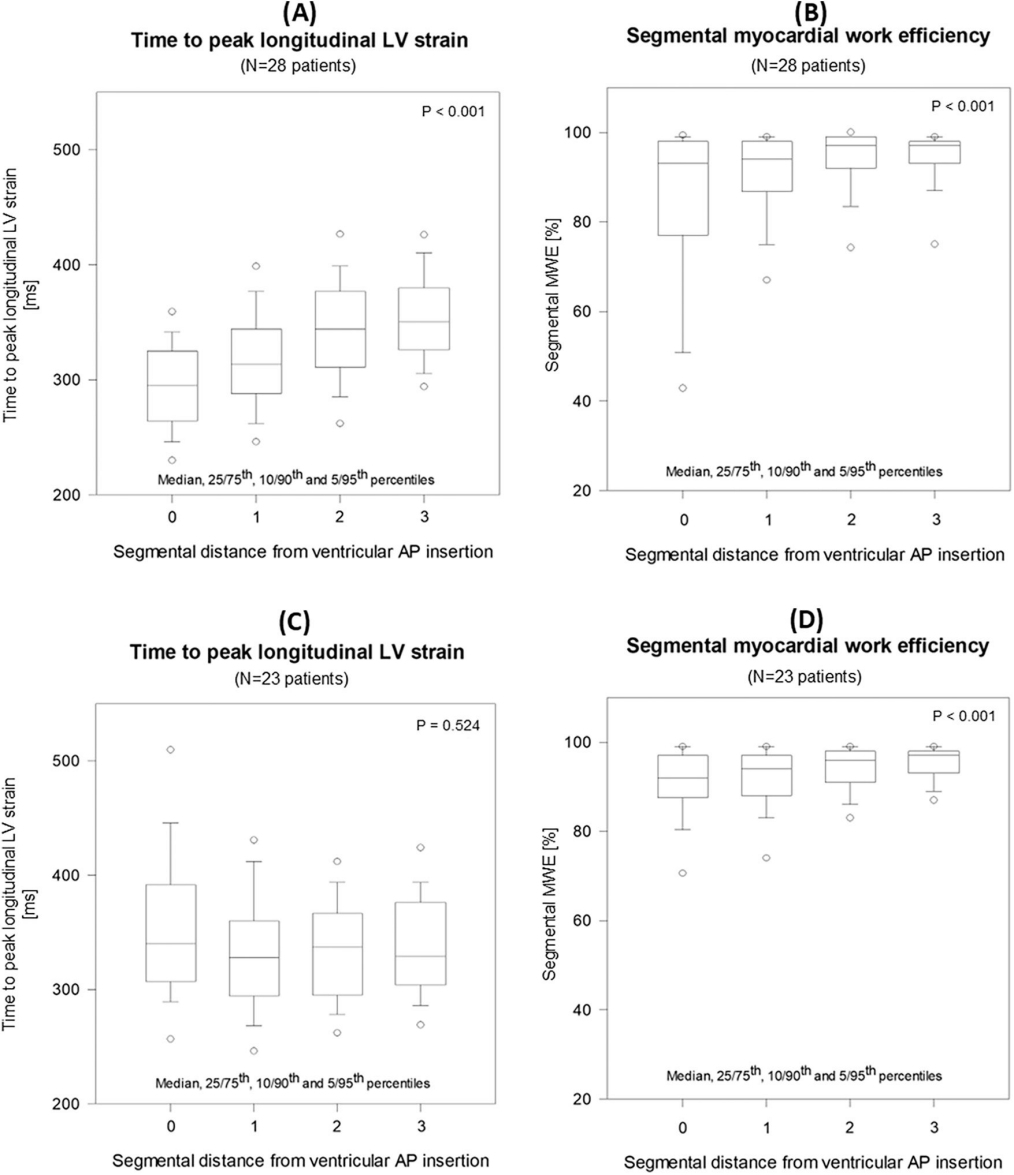
Segmental data and the distance from AP insertion. LV segmental time to peak longitudinal strain and myocardial work efficiency expressed as a function of the distance from ventricular AP insertion in patients with a single left free wall or paraseptal AP before [**(A)** and **(B)**; *N* = 28] and after [**(C)** and **(D)**; *N* = 23 patients] ablation. *X* axis denotes the respective segmental distance from ventricular pathway insertion (0, identical segment; 1, neighboring segment; 2, one interposed segment; 3, two interposed segments (see Figure 2*)). Analysis limited to patients with single left free wall and paraseptal pathways. The difference between individual segments was evaluated using ANOVA. AP, accessory pathway; LV, left ventricular; NS, nonsignificant. Figure 6 was created in the SigmaPlot statistical software (see the statistics section), labels were added in MS PowerPoint.

**Figure 7:**
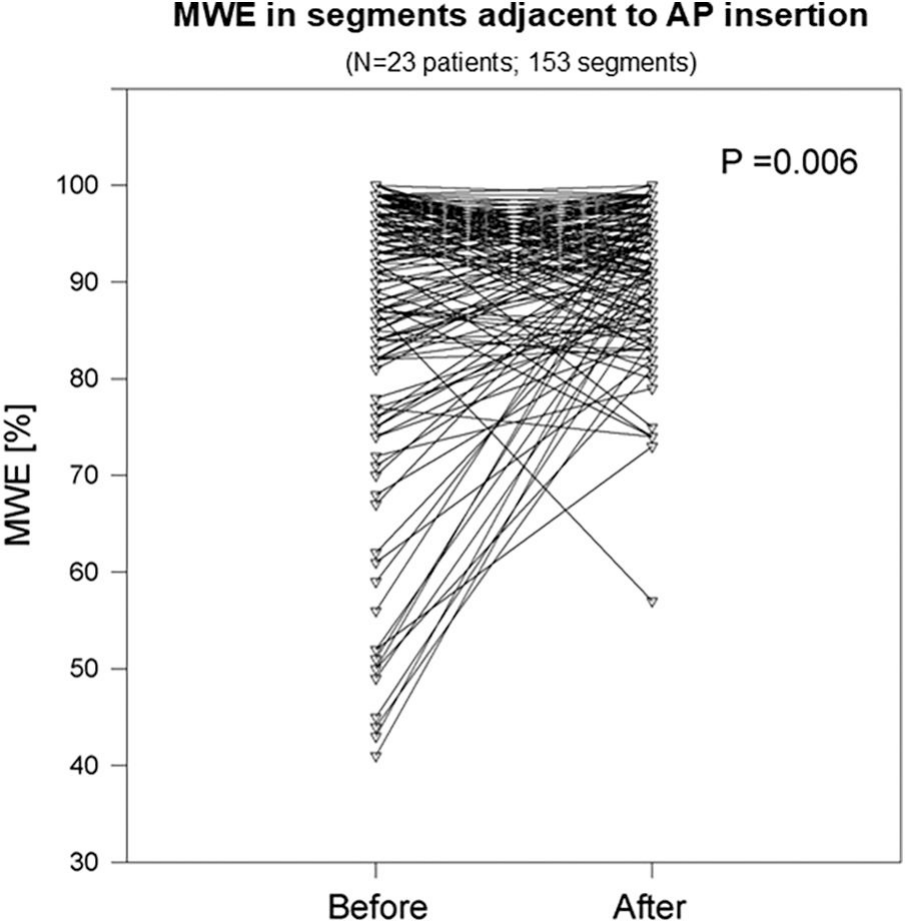
Myocardial work efficiency in segments (*N* = 23 patients, *N* = 153 segments) adjacent to ventricular AP insertion (segments 0 and 1, see methods) before and after ablation. AP, accessory pathway; MWE, myocardial work efficiency. Figure 7 was created in the SigmaPlot statistical software (see the statistics section), labels were added in MS PowerPoint.

Analysis was performed in 31/37 with successful AP ablation. There was a significant decrease in diastolic blood pressure as compared to the pre-ablation evaluation from mean (SD) 74.3 (5.3) to 62.0 (6.8) mmHg, *P* < 0.001, without a change in the systolic blood pressure [115.7 (15.6) vs. 116.8 (9.6) mmHg]. After ablation, there was a non-significant decrease of both global GWI and GCW potentially associated with the decrease in diastolic blood pressure. Global LV MWE increased along with a decrease in MWW (Table 5). There was no acute significant change of LV ejection fraction. AP ablation led to normalization of segmental pressure-strain loops (Figure 5). Patients with single LV free wall and paraseptal APs (*N* = 28) showed at baseline a significant correlation between both the time to peak longitudinal segmental LV strain and segmental LV MWE, resp., and the distance of the analyzed segment from the AP ventricular insertion (Figure 6A,B). Segmental MWE increased from median 93.0% at AP location to 97.0% in distant segments (*P* < 0.001). After ablation (*N* = 23), time to peak longitudinal segmental LV strain was equally distributed among LV segments, whereas segmental LV MWE still showed lower MWE in segments adjacent to AP location (Figure 6C,D). However, the standard deviation of segmental LV MWE decreased significantly from mean 9.4 (SD 5.1) before to 5.9 (SD 2.5) % (*P* < 0.001) after ablation as a measure of more homogenous MWE distribution. AP ablation carried a significant increase in LV MWE specifically in segments adjacent to the ventricular AP insertion (segments 0 and 1, *P* = 0.006, Figure 7) with 31/153 (20.3%) segments showing an increase of >10 points. No significant change was observed in remote segments (segments 2 and 3, *P* = 0.634). A minority of segments showed a decrease in MWE of >10 points after ablation with equal distribution between segments 0 and 1 (11/153, 7.2%) and segments 2 and 3 (22/311, 7.1%, p NS). In comparison to controls, WPW patients after ablation still showed lower LV ejection fraction (mean 56.8 vs. 59.3%, *P* = 0.037) as well as global LV MWE (median 94, IQR 92–95 vs. median 95, IQR 94%–96%, *P* = 0.023).

**Table 5:** WPW patients before and after AP ablation.

Parameter	Before (*N* = 31^a^)	After (*N* = 31^a^)	*p*-value	Power of the test
LV EF [%] *mean (SD)*	55.3 (6.2)	56.8 (4.4)	0.072	0.439
Global longitudinal LV strain [%] *mean (SD)*	−19.2 (2.5)	−18.9 (2.0)	0.980	0.050
Peak longitudinal LV strain dispersion [ms] *mean (SD)*	39.7 (7.9)	37.4 (9.9)	0.489	0.104
Global LV MWI [%mmHg] *mean (SD)*	1,804.0 (377.7)	1,702.6 (257.2)	0.336	N/A
Global LV MCW [%mmHg] *median (IQR)*	2,155.5 (1,907.8–2,439.0)	2,035.0 (1,859.0–2,256.3)	0.225	N/A
Global LV MWW [%mmHg] *mean (SD)*	179.0 (99.8)	121.9 (46.8)	0.004	0.864
Global LV MWE [%] *mean (SD)*	91.8 (3.7)	93.6 (2.1)	0.013	N/A

a Patients who underwent successful AP ablation.

AP, accessory pathway; EF, ejection fraction; IQR, inter-quartile range; LV, left ventricle/ventricular; MCW, myocardial constructive work; MWE, myocardial work efficiency; MWI, myocardial work index; MWW, myocardial wasted work; SD, standard deviation; WPW, Wolff-Parkinson-White.

### 3.6 Interobserver variability

**Figure 8:**
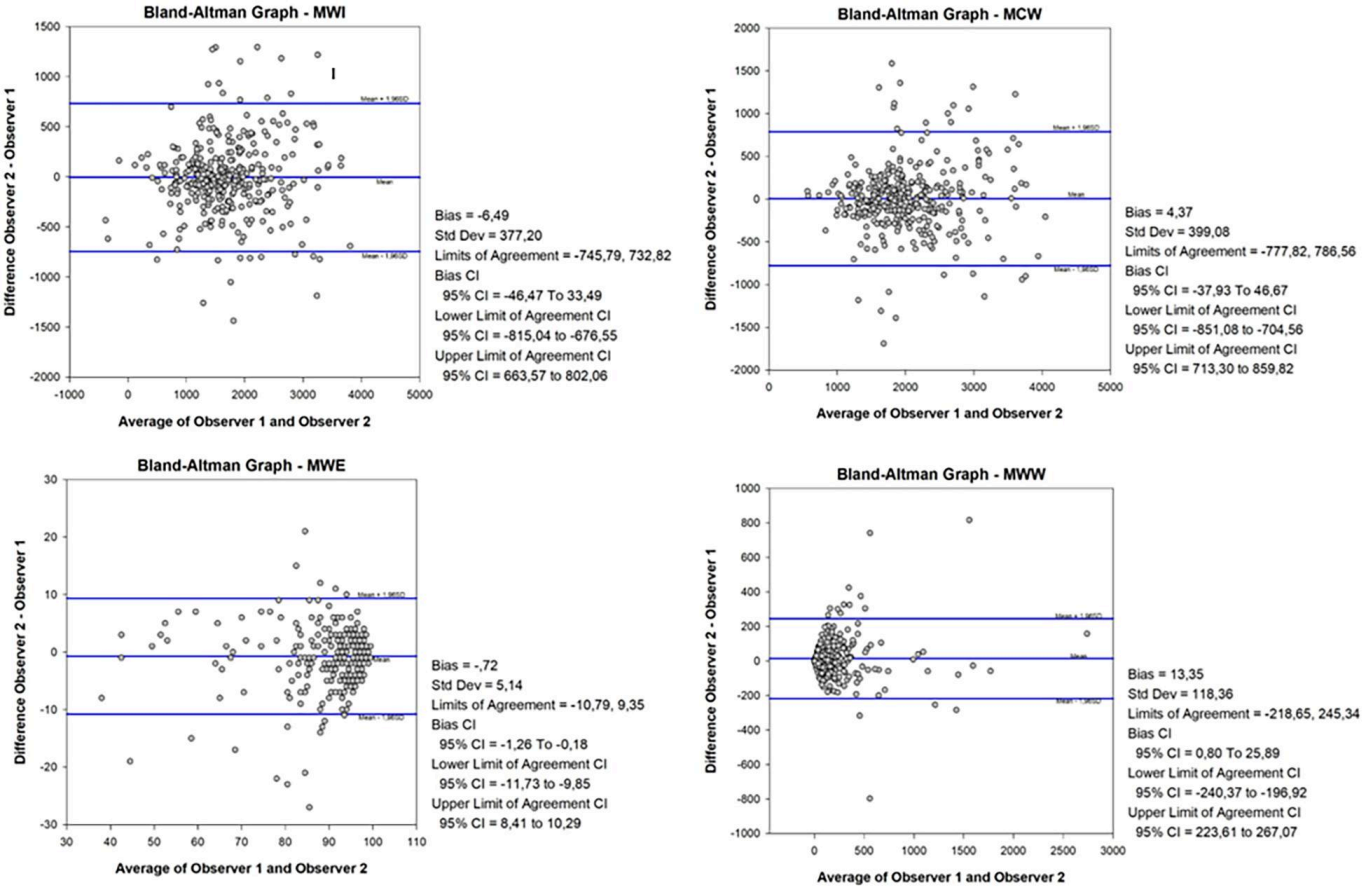
Bland-Altman plots describing inter-observer variability in segmental myocardial work parameters. CI, confidence interval; MCW, myocardial constructive work; MWI, myocardial work index; MWE, myocardial work efficiency; MWW, myocardial wasted work; Std Dev, standard deviation. Figure 8 was created in the SigmaPlot statistical software (see the statistics section), labels were added in MS PowerPoint.

Interobserver variability was evaluated for segmental MWI, MCW, MWW and MWE calculations and confirmed clinically acceptable interobserver differences within 2 standard deviations for the majority of individual measurements (Figure 8). Coefficient of variance for individual variables (MWI, MCW, MWW, MWE) is summarized in Table 6.

**Table 6:** Inter-observer coefficient of variance.

Data	MWI	MCW	MWW	MWE
Segmental data [%]	22.3	19.7	64.8	5.6
Global data [%]	9.5	7.2	18.9	1.6

MCW, myocardial constructive work; MWE, myocardial work efficiency; MWI, myocardial work index; MWW, myocardial wasted work.

## 4 Discussion

This Study focuses on the evaluation of MW in patients with WPW syndrome/pattern and its merit in clinical practice. The data demonstrates inferior MWE as compared to non-preexcited controls as well as its acute improvement in segments adjacent to AP location early after catheter ablation. Moreover, a specific pattern of LV myocardial discoordination resembling to a certain degree the so-called classic pattern dyssynchrony (9), as described in patients undergoing cardiac resynchronization therapy, has been reported in WPW preexcitation (1) with respective components of early segmental contraction at the ventricular AP insertion along with corresponding pre-stretch of remote segments and rebound stretch in the early activated area. As a consequence, LV MWE is specifically decreased in segments immediately adjacent to the ventricular AP insertion site.

Non-invasive MW evaluation has been so far used in adults (10–14) and children (15–18) in various disease entities as well as to define normal limits (19–22). One recent study has described MW in young patients with WPW preexcitation. The authors found that MWW is increased and MWE reduced in children with WPW, even if LV ejection fraction and global longitudinal strain were normal, thus showing the potential of MW analysis as a sensitive tool for the evaluation of myocardial function (23). However, the reported study did not compare the data before and after ablation and also does not report segmental MWE values and their relationship to AP location.

WPW patients were found at increased risk for LV mechanical discoordination and pathologic LV remodeling, and patterns of disturbed LV mechanical activation have been described in the past (1, 2). Our study goes farther in quantifying the degree of both global and segmental MW inefficiency and its relationship to the AP location and degree of preexcitation. Thus, it carries further information about the potentially negative impact of long-lasting ventricular preexcitation on LV function. LV MW inefficiency correlated closely with the degree of preexcitation as measured by either simple QRS duration or more precisely as the interval between earliest delta wave onset and R peak in lead V6 providing a simple possibility to detect patients at potential risk. AP location was not identified as an independent predictor of MWE beyond the degree of preexcitation, but the analysis was underpowered for right-sided pathways. In a recently published literature review (24) reporting on both pediatric and adult patients with manifest preexcitation-related cardiac dysfunction right septal and right free-wall APs were most commonly associated with cardiomyopathy. Degree of preexcitation has, however, not been specifically studied as a predictor.

Young patients with an asymptomatic WPW pattern are nowadays frequently detected by obligatory pre-participation screening including 12-lead ECG in many countries. Electrophysiologic evaluation is indicated/considered in patients with the asymptomatic WPW pattern as part of the risk stratification regardless of the non-invasively obtained AP properties. Prophylactic catheter ablation is indicated based on the antegrade conduction capacity of the AP to avoid life-threatening arrhythmias in case of atrial fibrillation with rapid atrioventricular conduction (25). Catheter ablation may also be considered in patients with low-risk asymptomatic pre-excitation as a IIb indication (26). The authors believe that the individual decision to ablate a low-risk AP may be supported by the finding of significantly decreased global MWE and such supportive indication may be discussed with the patient/legal guardians although not validated by longitudinal outcomes or part of the management guidelines at this point.

Catheter ablation has been found to improve but not fully normalize mechanical segmental LV function in the short-term. This is likely associated with long-lasting pathologic remodeling of underloaded segments close to the ventricular AP insertion site with corresponding hypotrophy and ultrastructural changes (27). Evaluation after ablation took place 16–24 h after intervention, which is probably not enough time to achieve complete reverse remodeling in underloaded segments close to the ventricular AP insertion site affected by hypotrophy and ultrastructural changes (1, 27). A longer follow-up would likely be needed to see complete reverse remodeling with full normalization of MW and LV ejection fraction as reported elsewhere (1). Published data (24) have shown that reverse remodeling may take several months in patients with significant preexcitation-associated LV dysfunction with single reported cases of irreversible myocardial changes.

In this study including only patients with well-preserved systolic LV function, changes in global ventricular mechanics before and after ablation are relatively minor. However, prior to ablation one fourth of the patients had global LV MWE of less than 90.5% (Table 2) due to significantly decreased MWE in segments adjacent to ventricular AP insertion (Figure 7), a pathology that may already translate into decreased maximum exercise capacity and play a significant role e.g., in competitive sports. Decreased exercise capacity with improvement after manifest AP ablation has been described in two recent pediatric studies (28, 29) and may be focus for further research. A highly significant increase in MWE in LV segments adjacent to AP insertion was noted in our cohort being in favor of the positive ablation effect.

No significant myocardial work index and MCW changes were found. These parameters are dominated by the majority of LV segments performing effective work. On the contrary, MWW and the derived MWE parameter reflect those few segments close to the AP insertion not contributing to pump function (with lengthening during systole and shortening against closed aortic valve). The relative magnitude of change in MWW and MWE is thus much higher after AP ablation and more likely to reach statistical significance.

### 4.1 Limitations

This study was designed to evaluate acute MW changes and did not include longer follow-up to assess reverse myocardial remodeling as a consequence of improvement of ventricular electromechanical synchrony after AP ablation. However, the association of dyssynchronous ventricular activation in WPW with pathologic ventricular remodeling and mid-term reverse remodeling after AP ablation has been described in prior publications (1, 2). Segmental myocardial function at the site of AP ablation may be influenced by transient myocardial edema and local inflammation in the short term. Such changes may rather prevent acute improvement of segmental MWE than the opposite. In this study MWE increased, however, significantly in AP adjacent segments. The method of MW calculation used in this study is non-invasive, load-independent (5, 6) and may have higher sensitivity than ejection fraction or global longitudinal strain for the detection of LV dysfunction (15, 23). It does not take, however, into account other variables such as geometric parameters of the ventricle etc. (10). In the studied patient group this limitation may, however, play a minor role as patients had a structurally normal heart and there were no LV volume changes between the pre- and post-ablation study. After ablation there was a significant decrease in diastolic blood pressure potentially affecting MWI, MCW and MWW calculations. However, MWE as a relative measure of work efficiency should not have been affected. Also, blood pressure changes could not be tracked in the course of the echocardiographic evaluation itself and might have further influenced the results. Further, high-quality echocardiographic images are required in order to be able to perform speckle tracking echocardiography and hence the MW analysis (7). Obtaining such images may require additional time and may hinder the use of this method as part of routine assessment of cardiac function. Our study was focused on LV MW and the relation between the AP insertion site and segmental MWE was studied only in paraseptal and left free wall APs to be able to clearly specify the site of earliest LV electrical activation and its relation to segmental MW disturbance. To perform such study in right-sided APs would necessitate using right ventricular MW analysis as described in the study of Butcher et al. in 2020 (11). The presented study also included a low number of patients with right free wall APs to allow for a more in-depth analysis. The evaluating echocardiographers were not strictly blinded to the AP location although there were not members of the electrophysiology team. However, quantitative global and segmental MW analysis is mainly an automatic ECHOPAC^TM^ software driven analysis not influenced by the evaluator beyond manual adjustment of LV wall detection and proper positioning of mitral and aortic opening and closing markers. The arrhythmia burden potentially affecting the pre-ablation measurements was not specifically evaluated, but none of the patients had an incessant tachycardia or running arrhythmia immediately prior or during the pre-ablation evaluation. Additionally, we did not acquire exercise stress testing data before and after ablation to correlate the degree of global LV MWE with maximum oxygen consumption. And lastly, total sample size of this study is modest but comparable to the only published study on MW in pediatric WPW patients (23). A statistical power analysis was, however, performed to support the main conclusions. Although inter-observer agreement was not ideal for both the segmental and global MW parameters, the coefficients of variation for MWE data (Table 6) are significantly lower than the differences observed (Tables 2 and 5, Figures 5B,D and 6). Specifically, LV segments adjacent to ventricular AP insertion and displaying low MWE show a major improvement far beyond the reported inter-observer variance (Figure 7). Although a minority of segments in this Figure shows a decrease in segmental MWE after ablation, the paired t-test statistic still confirms a robust general improvement. We have specifically reviewed the echocardiographic images of segments showing a decrease in MWE values of >10 points and have found no systematic reason other than inferior image quality in the segments affected.

## 5 Conclusions

Ventricular preexcitation induces significant LV MW inefficiency in segments adjacent to AP which correlates with the degree of preexcitation and partially improves but does not fully normalize early after ablation. Such acute change may translate into longer-term complete reverse remodeling. The amount of wasted work in asymptomatic preexcitation may be useful for assessing the potential for pathologic LV remodeling or discussing the indication for prophylactic ablation.

## Data Availability

The raw data supporting the conclusions of this article will be made available by the authors, without undue reservation.
